# Gadolinium: pharmacokinetics and toxicity in humans and laboratory animals following contrast agent administration

**DOI:** 10.1007/s00204-021-03189-8

**Published:** 2022-01-08

**Authors:** Julie Davies, Petra Siebenhandl-Wolff, Francois Tranquart, Paul Jones, Paul Evans

**Affiliations:** GE Healthcare, Pollards Wood, Nightingales Lane, Chalfont St. Giles, UK

**Keywords:** Gadolinium, Gadolinium-based contrast agent, Toxicity, Nephrogenic systemic fibrosis

## Abstract

Gadolinium-based contrast agents (GBCAs) have transformed magnetic resonance imaging (MRI) by facilitating the use of contrast-enhanced MRI to allow vital clinical diagnosis in a plethora of disease that would otherwise remain undetected. Although over 500 million doses have been administered worldwide, scientific research has documented the retention of gadolinium in tissues, long after exposure, and the discovery of a GBCA-associated disease termed nephrogenic systemic fibrosis, found in patients with impaired renal function. An understanding of the pharmacokinetics in humans and animals alike are pivotal to the understanding of the distribution and excretion of gadolinium and GBCAs, and ultimately their potential retention. This has been well studied in humans and more so in animals, and recently there has been a particular focus on potential toxicities associated with multiple GBCA administration. The purpose of this review is to highlight what is currently known in the literature regarding the pharmacokinetics of gadolinium in humans and animals, and any toxicity associated with GBCA use.

## History and use of magnetic resonance imaging

**Fig. 1:**
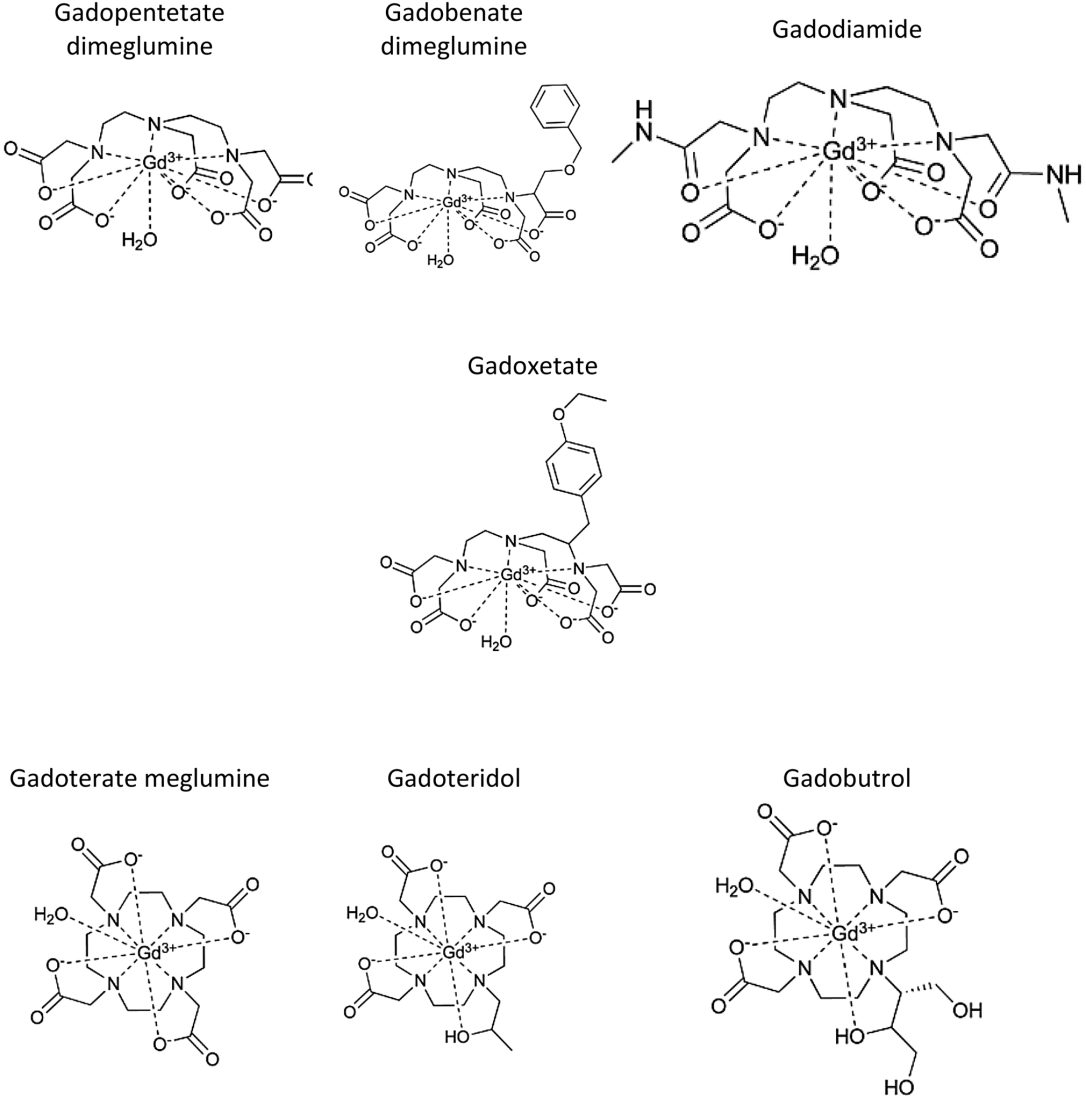
Chemical structures of commercially available gadolinium-based contrast agents

The 1940s marked the first use of nuclear magnetic resonance (NMR) and this was subsequently adapted using the interaction of magnetic gradients to develop a whole-body magnetic resonance imaging (MRI) scanner to image the human body (Bloembergen et al. 1948; Damadian et al. 1977). Hydrogen protons are abundant in the body due to high fat and water content, and they spin with random alignment. MRI employs the use of magnetic fields to force proton spin axes to undergo longitudinal alignment and when the field is turned off the protons return to their original spin axis, releasing radio frequencies, detected by coils used to construct MR images (Lauterbur et al. 1978). Soon after the discovery of MRI, the use of paramagnetic ions was investigated as a way of increasing the contrast and discernibility of images owing to the susceptibility of ions to external fields due to unpaired electrons (Lauterbur et al. 1978). Manganese, copper, chromium, ferric chloride and gadolinium-based contrast agents (GBCA) were initially used to assess the suitability of these ions as contrast agents (Runge et al. 1983). Gadolinium (Gd^3+^) showed the most promising enhancement effect of the paramagnetic ions tested. It was developed using a chelate (diethylenetriamine penta-acetic acid) to produce gadopentetate dimeglumine; the first clinically available GBCA, approved for use in multiple countries in 1988 as Magnevist, with 8 more GBCAs molecules being approved for worldwide use since then (Weinmann et al. 1984; Lohrke et al. 2016). Tens of millions of contrast enhanced MRI (CE-MRI) exams are performed annually around the world. Thirty tons of gadolinium metal ion were cumulatively administered to patients worldwide between 1988 and 1999 and this number has now exceeded 50 tons of gadolinium annually (Caravan et al. 1999). GBCAs utilise gadolinium as it creates a high magnetic moment which results in shortening of the T1 and T2 relaxation times of surrounding hydrogen protons, resulting in increased signal and improved contrast on MRI scans. Since the first clinical use of gadopentetate other GBCAs have been commercialised for clinical use, including gadoterate meglumine, gadoteridol, gadodiamide, gadobutrol, gadobenate dimeglumine and gadoxetate (Fig. 1) (Port et al. 2008). The success of GBCAs is in part because no suitable, non-invasive widely applicable alternatives exist, although some alternative metals have been used for example ferromagnetic particles. GBCAs were thought not to cross an intact BBB, so contrast enhancement of the brain can only occur if there is BBB perturbation as a result of disease, for example multiple sclerosis, cancer, or stroke. The use of GBCAs is not limited to the central nervous system (CNS) as they can also detect vascular permeability associated with non-CNS lesions and are frequently employed in the detection and staging of cancer as well as vascular, cardiac and joint imaging (Lohrke et al. 2016). However, recent research has raised safety concerns over the use of these agents (Wahsner et al. 2019). Even though many studies have shown gadolinium retention in human and animal tissues following GBCA administration, to date there is no evidence of clinical significance associated with the use of these agents in patients with normal renal function. Thus, if the clinical benefit outweighs any perceived risk, GBCAs could be used in diagnosis (Marks et al. 2021).

## Classes of gadolinium-based contrast agents

GBCAs are administered intravenously in contrast-enhanced MRI CE-MRI and distribute in the blood and into the extravascular space. Free gadolinium ions are highly toxic when not chelated due to interference with calcium ion channels resulting in toxicity, including neurotransmitter inhibition, muscle contraction and mitochondrial dysfunction (Bourne and Trifaró 1982; Marie Caille et al. 1983; Quarles et al. 1994; Feng et al. 2010). Thus the need to make this paramagnetic ion suitable for in vivo MRI by chelating the free gadolinium with a chelator (organic ligand), which reduces toxicity by making the Gd^3+^ unavailable to interact with tissues, facilitating quicker elimination and reducing biotransformation and accumulation (Cacheris et al. 1990; Chang 1993). Free gadolinium which is chelated is known as a GBCA and the different classes of GBCAs have inherent differences in physical properties which can be categorized into structural subtypes, charge, thermodynamic stability and relaxivity. GBCAs are categorised as linear or macrocyclic agents, dependant on the type of chelate (a polyaminocarboxylate ligand) which binds the Gd^3+^. Linear agents have a chain-like chelate structure which tightly grasps the Gd^3+^, whereas macrocyclic agents have a closed, ring like chelate structure which functions as a cage. The strength of the Gd^3+^ association with the chelate is measured using conditions which potentially mimic GBCA intra- and extracellular distribution in vivo to determine the stability and ability to undergo transmetalation (the replacement of gadolinium within the chelate by another ion, releasing Gd). This demonstrates that whilst all approved agents bind Gd very tightly with dissociation constants that favour the chelated structure, a cage like structure as found with macrocyclic GBCAs, are less labile over time and in different conditions (Table 1). The physicochemical properties of the various approved agents are listed in Table 1.

**Table 1:** Characteristics of MR GBCAs

Brand name	Chemical	Structure	Thermodynamic stability (*K*_therm_)	Conditional stability (Log *K*_cond_ at pH 7.4)	Kinetic stability in acidic conditions (HCl, pH1.2) at 37 °C; dissociation half-life	T1 relaxivity in plasma at 1.5 T (L/mmol s)	Agent type	Excess chelate (%)
Magnevist	Gadopentetate (dimeglumine)	Linear ionic	22.5	17.7	< 5 s	4.1	Nonspecific extracellular	0.1
MultiHance	Gadobenate (dimeglumine)	Linear ionic	22.6	18.4	< 5 s	6.3	Liver	0
Eovist/Primovist	Gadoxetate	Linear ionic	23.5	18.7	< 4 s	6.9	Liver	0.5
Omniscan	Gadodiamide	Linear non-ionic	16.9	14.9	< 5 s	4.3	Nonspecific extracellular	5
Dotarem	Gadoterate (meglumine)	Macrocyclic ionic	25.8	19.3	4 days	3.6	Nonspecific extracellular	0
ProHance	Gadoteridol	Macrocyclic non-ionic	23.8	17.1	4 h	4.1	Nonspecific extracellular	0.1
Gadavist/Gadovist	Gadobutrol	Macrocyclic non-ionic	21.8	14.7	18 h	5.2	Nonspecific extracellular	0.1

Port et al. (2008), Ringe et al. (2010), McDonald et al. (2018), Le Fur and Caravan (2019), Rudnick et al. (2021)

## Stability of gadolinium-based contrast agents in vitro and in vivo

The association of Gd^3+^ with its chelate functions as an equilibrium and is dependent on kinetic inertia and thermodynamic stability. The kinetic inertia relates to the rate of Gd^3+^ release, whereas the thermodynamic stability relates to how much of the Gd^3+^ is released under certain, stable conditions. The kinetic inertia of Gd^3+^ and its association with the chelate are dependent on the dissociation rate (the rate at which the Gd^3+^ dissociates from the chelate) and the extent of in vivo Gd^3+^ release is dependent on the elimination rate. If the dissociation rate is slower than the time taken for the GBCA to be eliminated from the body, then Gd^3+^ release is irrelevant as it is excreted before this can occur (classified as high kinetic inertia) (Frenzel et al. 2008). Whereas with a faster dissociation rate and a slower elimination rate of GBCAs, Gd^3+^ can be released in vivo (low kinetic inertia), potentially resulting in gadolinium retention in certain tissues. K_therm_ represents the stability of the deprotonated gadolinium complex (dissociation equilibrium) and K_cond7.4_ represents the conditional stability at physiological pH. Ionic GBCAs generally have higher stability constants than non-ionic GBCAs due to negatively charged atoms binding more strongly to Gd^3+^ than uncharged atoms (Lauffer 1987). The stability of these agents is not only important with regard to release of Gd^3+^ but also for the risk of transmetalation and endogenous metal sequestration by the chelate. This process has the potential to appropriate important physiological metals like iron, calcium or zinc into the chelate as well as competitive binding of Gd^3+^ by endogenous anions like PO_4_^3−^ and CO_3_^2−^, although transmetalation is considered the most important route for Gd^3+^ release (Cacheris et al. 1990). With some GBCAs excess chelate is included in the formulation to capture any released gadolinium, but this may also have the unintended consequence of capturing endogenous metals (Prybylski and Jay 2017). The stability of these GBCAs in vivo can be inferred by in vitro stability studies. Incubation of GBCAs (1 mmol/L) with human serum over 15 days (pH 7.4 and 37 °C) demonstrates the propensity of non-ionic linear GBCAs to be the least stable with increased release of Gd^3+^, while ionic linear agents were found to be more stable, and macrocyclic agents the most stable, with negligible Gd^3+^ release (Frenzel et al. 2008). However, there are some limitations when considering the relevance of such in vitro studies to the in vivo paradigm, where there is a highly dynamic situation in terms of distribution to different compartments, rapid elimination from the blood and the potential for enzymatic processing. Other factors may also influence the stability of the GBCA including elimination time and phosphate concentrations, which can be elevated in renal insufficiency. Studies using addition of phosphate to human serum to partially mimic the increased phosphate seen in renal insufficiency results in an increase in the release of Gd^3+^ from non-ionic linear agents by 15–16% compared to incubation without phosphate (Frenzel et al. 2008).

## Pharmacokinetics of gadolinium-based contrast agents in humans and animals

In the clinic GBCAs are administered intravenously (i.v.) and thus are 100% bioavailable as this bypass’s absorption barriers (such as the gut) for direct delivery into the circulation. GBCAs rapidly distribute from the intravascular to the interstitial compartment, which combined forms the extracellular compartment of initial distribution, with an added, delayed intracellular component observed for liver specific agents (Chang 1993). The short-term distribution and clearance of administered GBCAs has been well studied in humans and animals, and this has determined there are differences in the pharmacokinetics between humans and animals for certain agents (Table 2).

**Table 2:** Pharmacokinetics in vivo in humans after intravenous administration

GBCA	Distribution half-life (min)	Elimination half-life (min)	Serum elimination half-life in renal impairment (h)	Injected dose eliminated within 24 h (%)	Elimination path	Renal clearance rate (mL/min/kg)	Plasma clearance rate (mL/min/kg)	Volume of distribution (mL/kg)	Protein binding
Magnevist (gadopentetate dimeglumine)	12 ± 7.8	96 ± 7.8	Mild; 2.6 ± 1.2 Moderate; 4.2 ± 2.0 Severe; 10.8 ± 6.9	91 ± 13	Renal	1.76 ± 0.39	1.94 ± 0.28	266 ± 43	No
MultiHance (gadobenate dimeglumine)	5.04 ± 0.72 to 36.3 ± 4.32	70.2 ± 15.6 to 121.2 ± 36	Moderate; 6.1 ± 3.0 Severe; 9.5 ± 3.1	> 80%	Renal (93%), biliary (0.6–4%)	1.37 ± 0.12 to 1.73 ± 0.65	1.55 ± 0.17 to 2.22 ± 4.5	170 ± 16 to 282 ± 79	Weak
Eovist/Primovist (gadoxetate)	–	54.6–57	–	Not detected	Renal (50%), biliary (50%)	–	–	210	< 10%
Omniscan (gadodiamide)	3.7 ± 2.7	77.8 ± 16	–	95.4 ± 5.5	Renal	1.7	1.8	200 ± 61	No
Dotarem (gadoterate meglumine)	–	84 ± 12 (F), 120 ± 42 (M)	Moderate; 5.1 ± 1 Severe 13.9 ± 1.2	72.9 ± 17 (F), 84.4 ± 9.7 (M) (48 h elimination)	Renal	1.27 ± 0.32 (F), 1.40 ± 0.31 (M)	1.67 ± 0.17	179 ± 26 (F), 211 ± 35 (M)	No
ProHance (gadoteridol)	12 ± 2.4	94.2 ± 4.8	–	94.4 ± 4.8	Renal	1.41 ± 0.33	1.5 ± 0.35	204 ± 58	No
Gadavist/Gadovist (gadobutrol)	–	108.6 (79–127)	Mild/Moderate; 5.8 ± 2.4 Severe; 17.6 ± 6.2	> 90 (12 h elimination)	Renal	1.56 ± 0.18	1.78 ± 0.43	210 ± 20	No

NB: female (F), male (M)

Bayer HealthCare Pharmaceuticals (2018), Bayer HealthCare Pharmaceuticals (2011), Bracco Diagnostics (2010), Bracco Diagnostics (2013), GE Healthcare (2010), Guerbet (2017); Harpur et al. 1993; Staks et al. 1994; McDonald et al. 2018)

There is a clinical necessity to understand the dynamics of gadolinium distribution, particularly in certain patient populations. There are still unidentified mechanisms in osteoporosis, renal osteodystrophy, and BBB disruption and their effects on retention and transmetalation in humans (Abraham et al. 2008; Greenberg 2010; Ghio et al. 2011; Kanal 2016). A technique called inductively coupled plasma mass spectrometry (ICP-MS) has been used to measure gadolinium retention in the brain, bone, and skin of patients with a history of GBCA use, to better understand the dynamics of linear and macrocyclic agents in humans using post-mortem samples. There are obvious challenges to this technique in so far as it can only be used on ex vivo tissue samples, whereas indirect methods such as MRI to examine MR hyperintensity can be employed during non-invasive diagnostic imaging. Furthermore, ICP-MS can only detect the presence of gadolinium, it does not determine whether the form of the gadolinium is as a dissociated ion, chelated to the GBCA or bound to another molecule. Direct measurement of gadolinium demonstrates that gadolinium associated with macrocyclic GBCA use is cleared more rapidly from tissues than that with linear agents. The gadolinium present after macrocyclic administration is 306-fold lower in the brain, skin and bone of patients compared with linear GBCA use and this elimination has been shown to be biphasic with fast clearance in the first few weeks after administration, and slower clearance thereafter (Kobayashi et al. 2021; Strickler and Clark 2021). This elimination profile is likely a result of an initial, rapid, acute clearance of the majority of intact GBCA from the blood and extracellular fluid (ECF), followed by a slower component related to residual low levels of intact GBCA clearing from other compartments and/or dissociation of Gd^3+^ from the chelate (Kobayashi et al. 2021; Tweedle 2021).

The clearance route of GBCAs is primarily through glomerular excretion with a high fraction excreted in urine, without metabolic chemical modification. Approximately 90% of administered GBCA is excreted in the urine within 24 h in patients with normal renal function. There is, however, some slight degree of variation dependent on the class of GBCA which suggests more heterogenous and complex excretion in humans compared with animals, probably reflective of the wider biological diversity across the human population compared with any particular strain of laboratory animals. In renal impairment there are significant effects on the clearance of GBCAs, with circulating GBCAs being increased up to 12-fold not only impacting the elimination but most likely, the distribution (Cao et al. 2016b; Kartamihardja et al. 2016a). The elimination half-life of gadodiamide in patients with severe renal disease (glomerular filtration rate [GFR] of 2–10 mL/min) is 34.3 h ± 22.9, compared with 1.3 h ± 0.25 in those with no renal dysfunction, as a result of reduced clearance by poorly functioning kidneys (Joffe et al. 1998). Clearance data which extend past 24 h are more limited, but Gd^3+^ has been detected in urine long after GBCA administration which suggests the presence of a deep compartment in the body which can slowly release gadolinium over time (Lancelot 2016; Semelka et al. 2016a; Di Gregorio et al. 2018). Gadolinium has been shown to be detected in the bone of patients administered GBCAs up to 8 years post-dosing with gadodiamide exhibiting 4 times the gadolinium concentration compared with gadoteridol, although such data are difficult to compare directly due to many potentially confounding factors (White et al. 2006; Darrah et al. 2009). The differences in gadolinium retention may be partly based on the increased stability of macrocyclic and ionic agents (Tweedle et al. 1995; Frenzel et al. 2008; Pietsch et al. 2009).

In animals, as with humans, the general-purpose imaging GBCAs are ECF agents and are almost exclusively renally excreted without metabolic chemical modification. In rodents, general-purpose GBCAs which undergo renal excretion are excreted largely intact with 92–96% being excreted one hour post-dosing and more than 98% excreted in the urine by 24 h (Tweedle et al. 1995). Excretion of intact molecules demonstrates no known metabolism of GBCAs and in rats the half-life of GBCAs is approximately 20 min (Oksendal and Hals 1993; Caravan et al. 1999). Clearance from the blood follows first-order kinetics and a human equivalent dose in rats results in gadolinium below the limit of quantification (BLOQ) in the blood, 10 weeks post-dosing (Davies et al. 2021). Following injection of the liver specific imaging agent gadoxetate, 50% of the injected dose is excreted via the liver in humans, compared with a higher proportion (70%) in rats, which qualitatively is a similar species difference to that observed with gadobenate (0.6–4% in humans, 25–55% in rats, dogs, rabbits and monkeys) (Schuhmann-Giampieri et al. 1993, 2014; Lorusso et al. 1999; Eovist [package insert]). Gadobenate can also be used as a liver agent owing to this liver excretion; however, the inter-species difference in excretion profiles makes it less effective as an imaging agent than gadoxetate. The clearance half-life of GBCAs in mice is 5–6 min and in rats 18–20 min, and clearance of retained gadolinium is thought to be 1–3% per day (Tweedle et al. 1995). There are still gaps in our understanding of clinical pharmacokinetics including a full understanding of tissue distribution, transmetalation, trafficking and long-term excretion and distribution.

## Gadolinium distribution in humans and animals

### Gadolinium in the brain

The presence of retained brain gadolinium first gained widespread attention in a 2014 study which demonstrated hyperintensity in unenhanced T1-weighted images of the dentate nucleus and globus pallidus in those with a history of GBCA use with no history of renal impairment (Kanda et al. 2014). It was unclear if the hyperintensity was related to gadolinium, disease progression or other factors, but was subsequently confirmed to be gadolinium retention, with hyperintensity correlating with directly measured gadolinium concentrations (McDonald et al. 2015). Since then, other retrospective analyses have revealed similar signal changes after repeated doses of different classes of GBCAs (Kanda et al. 2015b; Quattrocchi et al. 2015; Radbruch et al. 2015; Ramalho et al. 2015; Cao et al. 2016a; Stojanov et al. 2016). The studies have been followed by others showing the presence of gadolinium years after linear and macrocyclic GBCA administration in post-mortem brain samples, concordant with unenhanced brain hyperintensities (Kanda et al. 2015a; Murata et al. 2016b; McDonald et al. 2017c). More recently, glioma patients who underwent CE-MRI before surgery showed gadolinium presence by ICP-MS in 57% of the glioma biopsies and 62% in normal appearing brain tissue present in the sample (Kiviniemi et al. 2019). This retention was also found in patients without BBB disruption and in non-diseased brain tissue (McDonald et al. 2017c). This has also been observed in paediatric cases after injection of gadodiamide and gadopentetate (Roberts et al. 2016a; McDonald et al. 2017c). In general the degree of gadolinium retention may be associated with the stability of the agents to some extent, with the more stable macrocyclic agents exhibiting less gadolinium retention compared with the less stable linear agents, but differences have been observed within sub-classes (e.g. linear compared with linear, and macrocyclic compared with macrocyclic) (McDonald et al. 2017b, 2018).

The advent of hyperintensity is possibly the most well studied phenomenon in terms of gadolinium retention in humans due to the non-invasive nature of MRI. Retrospective studies have been used to correlate hyperintensities detectable by MRI to history of GBCA usage. Patients with multiple sclerosis or brain metastases who had been exposed to at least 2 gadodiamide administrations showed a progressive increase in T1 hyperintensity in the dentate nucleus with unenhanced MRI (Errante et al. 2014)**.** This increase in T1 hyperintensity is correlated with linear GBCA administration and such correlation is generally not seen with macrocyclic agents (Kanda et al. 2014, 2015b; Radbruch et al. 2015, 2017). To correlate MRI hyperintensity with gadolinium concentration, ICP-MS was used to measure gadolinium in post-mortem brain samples in patients with a history of GBCA administration, and gadolinium was detected in all brain samples (Kanda et al. 2015a). This increase in T1 signal intensity was only seen in patients who had been given linear agents, and not macrocyclics. However, other studies have shown that gadolinium retention occurs after either linear or macrocyclic administration and there has been some evidence of hyperintensity with macrocyclic use (Murata et al. 2016a)**.** Notably, in patients with relapsing–remitting multiple sclerosis who were given repeated injections of the macrocyclic agent gadobutrol, there was an increase in T1 signal intensity detected in the dentate nucleus and globus pallidus, with greater hyperintensities where there were shorter intervals between gadobutrol administrations (Stojanov et al. 2016)**.** This is not the only example of hyperintensity associated with macrocyclic use, as 158 multiple sclerosis patients who had received either gadoterate or gadobutrol exhibited an increase in the dentate nucleus-to-pons ratio (gadoterate 0.0032 ± 0.0216, gadobutrol 0.0019 ± 0.0346) (Splendiani et al. 2018). The differences were not, however, significant but hyperintensity was found in around one-third of all patients who received at least 5 doses. This hyperintensity with gadobutrol administration has also been observed in patients with brain tumours, with a statistically significant dose-dependent enhancement seen in the dentate nucleus (Bjørnerud et al. 2017). Multiple, repeated administrations of macrocyclic agents did not result in hyperintensities with normal renal function, but was seen in those with renal impairment in the dentate nucleus (Lee et al. 2017). This finding of hyperintensity associated with macrocyclic usage, is not always found, and this discrepancy is not well understood and may be as a result of different scanners, field strength or imaging protocols. Additionally, it is not known which gadolinium species results in T1-weighted hyperintensity due to the possibility that only the MR visible forms are detected (McDonald et al. 2018)**.** Post-mortem samples of patients with normal renal function had 0.1–0.58 µg gadolinium per gram of brain tissue (globus pallidus, thalamus, dentate nucleus or pons) after having had at least 4 GBCA administrations over a period of 4 years, and there was a significant dose-dependent correlation with MR hyperintensity (McDonald et al. 2015). The gadolinium retention in the brain was localised to the vasculature, specifically the capillary endothelium and the neuronal interstitium and occurred in the absence of BBB perturbations, highlighting that a molecule previously thought unable to cross the BBB does enter the brain (McDonald et al. 2017c). As well as this retained gadolinium being associated with the vasculature, in those with severe renal disease it is also located in calcifications (Xia et al. 2010). In paediatric patients administered 3 doses of gadodiamide, gadolinium was again detected in the brain with the highest concentrations being in the pons and dentate nucleus. These gadolinium foci, similar to those seen in adults, were localised in the endothelium and in the neural interstitium, all in the absence of pathological neuronal findings (McDonald et al. 2017a). Paediatric patients with normal renal function who were administered multiple macrocyclic GBCA doses did not have increased T1 signal intensity in the brain on unenhanced scans (Tibussek et al. 2017). Additionally, multiple exposures to the linear agent gadoxetate was also not associated with an increase in signal in the dentate nucleus or globus pallidus (Conte et al. 2017). In patients with multiple sclerosis there has been an association with increased signal intensity in the dentate nucleus with lower verbal fluency scores; however, this has not been recapitulated and the presence of multiple sclerosis is a major confounder (Forslin et al. 2017). In patients with inflammatory bowel disease, there is no evidence of brain functionality changes in the presence of dentate nuclei and basal ganglia hyperintensity associated with repeated gadodiamide exposure (Quattrocchi et al. 2015). The nature of these studies is almost always small, single centre studies; however, they have provided considerable information on gadolinium retention; notably that T1-weighted unenhanced hyperintensities are almost always associated with repeated, linear GBCA administration. There is currently no evidence for any clinical effect of gadolinium retention or signal hyperintensity in the brain (Hoggard and Roditi 2017)**.**

T1-weighted unenhanced hyperintensity has been found not only in humans, but animals. The hyperintensity is concentrated in the dentate nucleus and globus pallidus of the deep cerebellar nuclei (DCN). And whilst this hyperintensity is correlated to gadolinium retention, it has not been associated with any histopathological changes in the brain (Lohrke et al. 2017; Smith et al. 2017; Davies et al. 2021). Robert et al. reported that the repeated administration of more than 30 times the human equivalent GBCA dose in rats resulted in significant T1 hyperintensity in the DCN of linear GBCA-treated animals, but not in rats treated with the macrocyclic class agent, gadoterate (Robert et al. 2016). Although the hyperintensities in the linear GBCA treatment groups were correlated with increased gadolinium concentration, they reduced significantly over time. A separate study concluded that the MR hyperintensity was increased with increased dose of a linear GBCA, but that there was no further increase after dose cessation (Robert et al. 2015). The linear GBCAs gadodiamide and gadobenate result in an increase in T1 signal compared to pre-contrast images of 35% and 30% respectively, after 50 mmol/kg GBCA administration over 26 days in rats. However, macrocyclic agents resulted in less hyperintensity with a T1 signal increase compared to pre-contrast images of 20% and 7% for gadobutrol and gadoteridol, respectively (McDonald et al. 2017b). However with a more clinically relevant dose, dogs administered 2–3 doses of gadodiamide had no differences in hyperintensity in the DCN or pons on unenhanced T1-weighted MR images, compared with pre-contrast images (Richter et al. 2020). The dentate nucleus and globus pallidus, the brain regions which for most studies appear to show the highest T1 hyperintensity are also areas where iron, copper and zinc concentrations are high, suggesting possible transmetalation by competition of the chelate for these metals, resulting in Gd^3+^ dissociation and subsequent retention (Lauffer 1987; Cacheris et al. 1990; Caravan et al. 1999; Rasschaert et al. 2018a; Minaeva et al. 2020). Even after a single dose of linear GBCAs, gadolinium retention measured by laser ablation-ICP-MS (LA-ICP-MS) in sheep brain was colocalised with iron, zinc and copper with specific accumulation in the DCN, but was BLOQ for a macrocyclic GBCA (Radbruch et al. 2019). The cage like structure of macrocyclic GBCAs may further protect gadolinium from dechelation, transmetalation and subsequent gadolinium retention in these metal rich areas of the brain, due to their increased stability. The thermodynamic stability constant of Gd-DTPA is 17.7 and for Fe-DTPA is 23.4, indicating that once transmetalated the iron chelate is more stable with Gd-DTPA being more prone to exchange gadolinium for iron (Port et al. 2008). However this is predicated on a high enough concentration of a labile pool of iron, which may be unlikely given that than iron is often associated with transporter proteins and unavailable for transmetalation (Rasschaert et al. 2020). It is also possible that gadolinium is concentrated in iron rich regions as they use the same access pathways such as transporter proteins such as transferrin, which gadolinium has been shown to bind to (Zak and Aisen 1988). Whilst it is clear that macrocyclic GBCAs are unlikely to result in hyperintensity in animals, this observation is undoubtedly more likely with linear GBCA administration with repeated, supraclinical doses (Cao et al. 2016a; Robert et al. 2016; McDonald et al. 2017b).

Gadolinium retention by chemical analysis in the brain of animals after GBCA administration has been well documented and was shown as early as 1995. Using an isotope of gadolinium (^153^Gd), measurement of radioactivity was performed in the brains of mice and rats administered gadoteridol, gadoterate, gadopentetate and gadodiamide up to 60 min post-dosing, but was BLOQ after 1 day (Tweedle et al. 1995). This study, however, used doses lower than a human equivalent dose and thus caution must be used in the interpretation of these data. In 2015 a study by Robert et al. gadolinium was detected in the cerebellum of healthy rats using ICP-MS after a cumulative dose of 12 mmol/kg (32 human equivalent doses when adjusted for differences in body surface area) of gadodiamide (3.66 ± 0.91 nmol/g) and gadoterate meglumine (0.26 ± 0.12 nmol/g) (Robert et al. 2015). Although cerebellar gadolinium was detected following gadoterate administration, there was no T1-weighted signal hyperintensity detectable by unenhanced MRI which was present with gadodiamide administration. This difference in hyperintensity by MR has been seen repeatedly with different classes of GBCAs. A cumulative dose of 50 mmol/kg of GBCAs revealed that linear agents exhibited more gadolinium retention in the brain (gadobenate; 4.7 µg/g, gadodiamide; 6.9 µg/g) than macrocyclic agents (gadoteridol; not detectable, gadobutrol; 1.6 µg/g) (McDonald et al. 2017b). Even within the same class the macrocyclic agents gadoteridol, gadoterate and gadobutrol, demonstrate differences in retention in the cerebellum (0.150 ± 0.022 vs. 0.292 ± 0.057 and 0.287 ± 0.056 nmol/g, respectively) and cerebrum (0.116 ± 0.036 vs. 0.250 ± 0.032 and 0.263 ± 0.045 nmol/g, respectively) (Bussi et al. 2018a, b). Given that dechelation of macrocyclic agents is thought to be more limited, this may be as a result of different clearance rates of the agents, possibly related to their physicochemical properties. We have previously assessed the clearance kinetics of gadolinium in the rat brain after a single human equivalent dose of gadodiamide. In this study, we evaluated discrete areas of the brain and characterised gadolinium clearance up to 20 weeks post-dosing. We found that 1 h post-dosing gadolinium levels were unsurprisingly at their highest (subcortex; 2.67 ± 2.17 nmol/g, left hippocampus; 5.73 nmol/g, left cortex; 4.29 ± 2.30 nmol/g, left hemisphere rest-of-brain 4.27 ± 2.00 nmol/g, right hemisphere rest-of-brain; 4.23 ± 1.99 nmol/g). There was a significant reduction 1d post-dosing in all brain regions analysed, which remained at very low levels up to 20 weeks post-dosing and less than 0.00004% injected dose (id) in all brain areas, but did not fall below the range of quantification (Davies et al. 2021). This demonstrates the rapid clearance from the brain, but with long-term retention of extremely small amounts of residual gadolinium. Rats administered 0.5 mmol/kg Gd[^14^C]DTPA-BMA (gadodiamide) showed rapid excretion, with only 1% of the injected dose remaining after 24 h (Kindberg et al. 2010). Quantitative whole-body autoradiography was used to measure the ^14^C, and gadolinium concentrations were measured by inductively coupled plasma atomic emission spectroscopy (ICP-AES) and inductively coupled plasma sector field mass spectrometry (ICP-SF-MS) to determine the association of chelate with gadolinium, to infer whether the ion was chelated or dissociated. The injected dose measured in the kidney, liver, lung, muscle, and skin were similar between the radioactivity measurements of the chelate and gadolinium concentrations, suggesting that up to 7 days the chelate is intact. This highlights that gadolinium measurement cannot be used as a measure of release from the chelate, as it may still be intact. This study did, however, use a slightly lower dose than would be administered in the clinic (0.62 mmol/kg, body surface area adjusted), and only extended to 7 days, when we know retention can occur even years post-dosing.

Models of multiple sclerosis using the experimental autoimmune encephalomyelitis (EAE) have been used to examine the impact of the disease on gadolinium retention by modelling some of the characteristics including brain inflammation, BBB disruption, myelin destruction, neuronal damage, and multifocal lesion formation. In a mouse model of EAE, magnevist administration (cumulative dose 20 mmol/kg) resulted in mild signal hyperintensity in the choroid plexus on unenhanced T1-weighted scans and whilst control animals also had detectable gadolinium in the brain (2.4 ± 0.6 μg Gd/g tissue), EAE animals had significantly more (5.3 ± 1.8 μg Gd/g tissue) (Wang et al. 2019). Gadodiamide administration (3.6 mmol/kg) in this model also results in increased gadolinium in the spinal cord compared to sham control animals when given during the onset of disease or in the chronic phase, but cerebrum and cerebellar concentrations were not different. This preferential retention may be as a result of massive demyelination in the spinal cord; however, this effect is transient and with longer washout times gadolinium retention decreases (Furlan et al. 2021).

As with gadolinium retention in other tissues, macrocyclic and linear agents exhibit different retention properties. Linear agents typically have slightly higher levels than macrocyclics, but brain retention of gadolinium has been demonstrated for all GBCAs, including macrocyclic agents (Bussi et al. 2018b). Transmission electron microscopy with energy-dispersive X-ray spectroscopy (TEM-EDS) has suggested the ultrastructural distribution of gadolinium is largely vascular and localised to the endothelium of capillaries on the luminal side of the BBB in rats (Davies et al. 2021). The foci observed were amorphous, suggesting that these were not crystalline deposits of inorganic salts but in fact likely to be organic. Additionally, rat studies have shown that all GBCAs can enter the CSF and that neither their structure or physicochemical properties affect their ability to penetrate and distribute within the CSF (Jost et al. 2017).

### Gadolinium in the bone

Osteoblasts can incorporate gadolinium into the bone matrix and replace calcium, functioning as a gadolinium reservoir (Abraham et al. 2008). Gadolinium is retained in the bone up to 8 years after the last GBCA administration and post-mortem studies show that the retention is highest in the bone compared with other tissues (Darrah et al. 2009; Murata et al. 2016a). Gadolinium retention with linear agents was 4.4 times higher in the bones compared with a macrocyclic agent, but both groups had the highest concentration of gadolinium in the bones compared with the other tissues (White et al. 2006; Kobayashi et al. 2021). The gadolinium concentration in the bone was found to be up to 13–23 times higher compared with the globus pallidus and dentate nucleus following either gadobenate or gadoteridol administration (Kobayashi et al. 2021)**.** Non-invasive techniques can be used to measure gadolinium retention in live patients. These techniques employ the use of an x-ray fluorescence analysis and this has allowed determination of gadolinium concentrations in the bone of patients administered gadobutrol to be 1.19 μg Gd/g bone mineral ± 0.73, with a significant correlation between gadobutrol dose and gadolinium concentration (Lord et al. 2018). Another study showed that retained gadolinium after Omniscan administration was higher (1.77 ± 0.704 µg Gd/g bone) compared with ProHance (0.477 ± 0.271 µg Gd/g bone) (Abraham and Thakral 2008). Presently, there are several post-marketing clinical studies underway which include a paediatric study looking at bone retention after repeated GBCA administration.

In preclinical studies, gadolinium retention after GBCA administration has consistently been shown to be highest in the bone (Murata et al. 2016a; Lohrke et al. 2017; Boyken et al. 2019; Davies et al. 2021). Some of the first evidence of bone retention was shown in mice and rats administered a radioactive isotope of gadolinium chelated as gadoteridol, gadoterate, gadopentetate and gadodiamide. All animals had gadolinium present in the femur up to 1-day post-dosing. After 14 days it was only detected in gadopentetate and gadodiamide groups in mice and gadoterate, gadopentetate and gadodiamide groups in rats (Tweedle et al. 1995). We have recently shown that after a single human equivalent dose of gadodiamide in rats, the tissue with the highest retention was the femur. Whilst there was an initial washout between 1 h and 1 day (81.69 ± 67.46 nmol Gd/g tissue and 18.46 ± 1.96 nmol Gd/g tissue, respectively), levels of gadolinium detected remained consistent up to 20 weeks (14.00 ± 0.06 nmol Gd/g tissue) post-dosing (Davies et al. 2021). Age has been shown to have an impact on bone gadolinium retention, with a higher concentration of gadolinium in the bone marrow of gadodiamide-treated juvenile rats compared with adults, but not noted in any other tissues analysed (Fretellier et al. 2019). Among animal models used to mimic BBB disruption that is seen in human CNS disease, one such model uses lipopolysaccharide (LPS) to stimulate sepsis in rats, and this results in an increase in bone retention with gadobenate (0.2 mmol/kg) administration compared to control animals (394.22 ng/g ± 62.6 and 292.52 ng/g ± 43.9, respectively). This increase in bone retention persisted in the sepsis model and was significantly increased compared to controls up to 3 weeks, but by 6 weeks had reduced to levels comparable to those in sham groups given GBCAs, demonstrating a transient effect of sepsis on bone retention in this model. Although LPS and sham animals had equivalent bone gadolinium at 6 weeks, gadolinium was still detectable and did not show much clearance over time, although the mechanisms for this difference in retention are unknown (Damme et al. 2020). This increased retention in the bone compared to other tissues has been hypothesised to be due to the bone serving as a reservoir or deep compartment for gadolinium, potentially resulting in the chronic and slow release of gadolinium over time as a result of the incorporation of gadolinium by osteoblasts into the bone matrix (Abraham et al. 2008; Darrah et al. 2009).

### Gadolinium in the skin

Gadolinium has been detected in the skin of patients with renal insufficiency in a variety of studies, using different techniques. Using scanning electron microscopy and energy-dispersive X-ray spectroscopy, the level of gadolinium has previously been measured and calculated as counts per second (cps). This technique has detected gadolinium in the skin of patients with a disease termed nephrogenic systemic fibrosis (NSF) which is associated with GBCA use. In 57 biopsies from 29 patients gadolinium concentration ranged from 1 to 2270 cps/mm^2^, and in another study with 20 patients ranged from 1 to 1666 cps (Abraham et al. 2008; Thakral and Abraham 2009). In a study with skin biopsies from patients with NSF, only 4 of 7 had measurable gadolinium (High et al. 2007). Within these positive cases, the number of gadolinium particles showed a large degree of variation with some samples not containing any gadolinium. In more quantitative studies gadolinium was measured by ICP-MS in patients with NSF and was found at high levels in the skin (320 μg Gd/g tissue), but was not detected in patients without NSF and a history of GBCA administration (Khurana et al. 2008). Additionally, when using affected and unaffected skin biopsies from patients with NSF, gadolinium concentrations were 71.4 ± 89.4 μg Gd/g tissue in affected tissues and 10.2 ± 19.9 μg Gd/g tissue in unaffected tissues (Christensen et al. 2011). In patients with normal renal function who had undergone 1 or more CE-MRI (gadodiamide or gadopentetate), no deposits of gadolinium were found in the skin (Boyd et al. 2008). Additionally, a patient who had a history of chronic kidney disease and exposure to GBCAs with no evidence of NSF, did not have any gadolinium deposition in the skin as measured by ICP-MS and spectroscopy (High et al. 2010). Although most studies have shown no gadolinium in the skin of patients administered GBCAs with normal renal function, one study found gadolinium present (14.5 ± 0.4 μg Gd/g) in a patient with normal renal function who underwent 61 CE-MRI, but at significantly lower levels than those seen in NSF samples (Roberts et al. 2016b). Since appropriate measures have been put in place, only 7 cases of NSF have been reported since 2008 (Attari et al. 2019).

Insoluble extracellular gadolinium foci have been identified in the skin of patients, with gadolinium foci observed extracellularly and intracellularly in macrophages, lysosomes and fibrocytes, potentially demonstrating in vivo transmetalation (High et al. 2007; Abraham and Thakral 2008; Thakral and Abraham 2009). These foci which are less than 1 µm in diameter have also been shown to be localised to areas of fibrosis, and in one example was found in the papillary dermis, underlying an actinic keratosis (High et al. 2007). Gadolinium which is retained in tissues may undergo macrophage phagocytosis, leading to association with cell bodies and intracellular localisation. Synchrotron x-ray fluorescence microscopy (SXRF) and extended absorption fine structure (EXAFS) spectroscopy analysis of a skin sample from a patient with NSF revealed the insoluble gadolinium foci were not associated with a chelator and thus was present as dechelated gadolinium (George et al. 2010). Clinical studies have focused on measurement of skin gadolinium in patients with NSF as most studies have not shown gadolinium in the skin of patients with normal renal function (High et al. 2007; Boyd et al. 2008; Khurana et al. 2008; Christensen et al. 2011).

Gadolinium retention in the skin of rodents has been demonstrated in multiple studies and can persist for up to 1 year post-dosing (Pietsch et al. 2009; Murata et al. 2016a; Lohrke et al. 2017; Bussi et al. 2018a, b; Fretellier et al. 2019). In a comprehensive study, rats were given a cumulative dose of 12.5 mmol/kg (20 times the human equivalent dose) of either a linear GBCA (gadodiamide, gadopentetate, gadobenate or gadoversetamide) or a macrocyclic GBCA (gadoteridol, gadoterate or gadobutrol) and skin biopsies were taken at various time points up to 364 days post-dosing (Pietsch et al. 2009). There were no macroscopic skin changes in any of the treated animals and ICP-MS measurement of gadolinium in the skin samples showed the highest retention with non-ionic linear treated animals (gadodiamide; 132 ± 23 and 72 ± 12 nmol Gd/g skin and gadoversetamide 47 ± 5 and 18 ± 5 nmol Gd/g skin, 35- and 364-days post-dosing, respectively). The ionic linear GBCAs also resulted retention up to one-year post-dosing but the levels of gadolinium were lower than those seen with non-ionic agents (gadopentetate; 36 ± 6 and 9 ± 2 nmol Gd/g skin and gadobenate; 7 ± 1 and 1.4 ± 0.4 nmol Gd/g skin, 35 and 364 days post-dosing, respectively). Following macrocyclic GBCA administration, gadolinium levels were far lower and close to the limit of detection (gadoteridol; 1 ± 1 and 0.08 ± 0.02 nmol Gd/g skin, gadoterate; 2 ± 1 and 0.22 ± 0.17 nmol Gd/g skin, gadobutrol; 2 ± 1 and 0.06 ± 0.03 nmol Gd/g skin, 35 and 364 days post-dosing, respectively). This temporal analysis shows that skin retention resolves in phases, with an immediate washout over days (macrocyclic GBCAs) or weeks (linear GBCAs) of what is most likely intact GBCA due to it being readily eliminable. In the chronic phase, there is a steady-state level of gadolinium, in which the concentration slightly decreases over time. For macrocyclic GBCAs, this decrease is enough to resolve the gadolinium concentration almost to control levels, but not for linear GBCAs where the concentration remains higher. Although the non-ionic, linear agents showed the highest retention at all timepoints, 1 year post-dosing the percent dose found in the skin was 0.08% and 0.02% for gadodiamide and gadoversetamide, respectively, effectively demonstrating a substantial clearance. The gadolinium concentrations in the skin of these animals can be correlated to the thermodynamic stability of the different agents, suggesting that GBCA lability in skin retention inversely correlates to the thermodynamic stability, although the physicochemical properties of the intact chelate (e.g. lipophilicity) will also play a role in distribution and retention in skin (Pietsch et al. 2009). Thermodynamic stability is measured by *K*_therm_ and *K*_cond_, with the latter being measured at physiological pH and possibly more relevant to stability in vivo. Interestingly, the macrocyclic agent gadobutrol has one of the lowest conditional stabilities among approved agents (Log *K*_cond_ 14.7), and even though gadobutrol *K*_therm_ is higher than the linear gadodiamide, gadodiamide has similar stability at physiological pH (Log *K*_cond_ 14.9) (Table 1). The other linear agents gadobenate and gadoxetate also have high stability at physiological pH, but gadoterate has the highest stability in terms of *K*_therm_ and *K*_cond_. This understanding of stability in vitro is a simplistic view, and in vivo stability is more complex in terms of stability and potential transmetalation. In a study with a cumulative dose of 50 mmol/kg (80 human equivalent doses) of gadodiamide, gadopentetate, gadobutrol or gadoteridol the gadolinium concentrations in the skin were significantly higher in gadodiamide-treated animals (1472 ± 115 nM Gd/g) compared with gadopentetate (80.8 ± 6.2 nM Gd/g), gadobutrol (1.1 ± 0.5 nM Gd/g) and gadoteridol (and 1.7 ± 0.8 nM Gd/g) treated groups (Lohrke et al. 2017). Whilst brain concentrations of both GBCA administered groups were similar, those in bone with gadodiamide were higher than seen with gadopentetate. This suggests that it is unlikely that recirculation from the bone reservoir (which is higher in the gadodiamide group) which may be released into the blood after bone reabsorption and remodelling, contributes to skin concentrations, which is similar between linear agents. In a recent study, Bussi et al. demonstrated that after repeated administration (12 mmol/kg cumulative dose) with macrocyclic GBCAs there were differences in gadolinium skin concentration (gadoteridol; 0.400 ± 0.112 nmol Gd/g skin gadoterate (Dotarem); 0.660 ± 0.202 nmol Gd/g skin, gadoterate (Clariscan); 0.688 ± 0.215 nmol Gd/g skin and gadobutrol; 0.999 ± 0.442 nmol Gd/g skin) (Bussi et al. 2020). The same authors have also shown that repeated macrocyclic GBCA administration (12 mmol/kg; gadoterate, gadobutrol and gadoteridol) result in no skin gadolinium 28 days post-dosing; however, the techniques employed here were not as sensitive and the limit of quantification for these studies was 1 nmol Gd/g (Bussi et al. 2018b). Finally, sex and age of rats has been shown to have no impact on gadolinium retention or lesion formation (Fretellier et al. 2019).

### Gadolinium in the other organs

Whilst the main focus of research into gadolinium distribution following GBCA administration has focused on the brain, skin and bone, it has also been studied in the musculoskeletal system, nerves and vasculature of patients administered GBCAs (Sanyal et al. 2011; Murata et al. 2016a). Phase IV studies are currently ongoing which aim to assess and understand in more detail gadolinium retention associated with GBCA usage (McDonald et al. 2018).

Whilst most nonclinical studies have focused on measurement of gadolinium in the brain, skin and bone, there is evidence of gadolinium retention in other organs. Tweedle et al. measured a radioactive isotope of gadolinium incorporated into gadoteridol, gadoterate, gadopentetate and gadodiamide (Tweedle et al. 1995). Gadolinium was detected in the blood, heart, lungs, liver, kidneys, spleen, GI tract and urine of all mice 60 min post-dosing after a dose of 0.48 mmol/kg. After 14 days post-dosing no gadolinium was detected in any of these organs with gadoteridol, and was only found in the liver, kidneys, and GI tract with gadodiamide and gadopentetate. A more comprehensive assessment was done in rats (0.1 mmol/kg), assessing more organs. The macrocyclic agents gadoteridol and gadoterate demonstrated rapid washout and in most organs was not detected 14 days post-dosing. There was residual gadolinium with the linear agents in the liver, kidneys, testes, stomach, and intestines. After a single human equivalent dose of gadodiamide in rats, gadolinium was detected 20 weeks post-dosing in the kidney (4.64 ± 1.21 nmol Gd/g tissue), liver (0.78 ± 0.25 nmol Gd/g tissue), lung (1.16 ± 0.25 nmol Gd/g tissue) and testes (0.22 ± 0.04 nmol Gd/g tissue), although all of these organs demonstrate significant washout over time (Davies et al. 2021).

## Entry of gadolinium-based contrast agents into the human and animal brain

The BBB functions to prevent the entry of pathogens, cells, and blood-derived components into the brain. BBB disruption can occur in a variety of neurological disorders including, multiple sclerosis, stroke and brain tumours, and this break down allows the entry of substances into the brain, that could otherwise not cross the BBB (Zlokovic 2011)**.** CE-MRI with GBCAs has been used for a plethora of CNS disorders associated with BBB perturbation to allow optimal imaging of the brain (Kanal and Tweedle 2015)**.** GBCAs are were thought to be incapable of crossing an intact BBB; however, that assumption has been challenged due to the presence of gadolinium with an intact BBB. Whilst it is still relatively unknown exactly how GBCAs permeate the BBB, some studies have elucidated several possible mechanisms for GBCA entry into the brain parenchyma, including via the blood–CSF barrier and the perivascular system (pial–glial pathway) as well as directly across the intact BBB. The blood–CSF barrier is comprised of the choroid plexus, which is a single layer of epithelium comprising an extensive capillary network linked by apical tight junctions (Engelhardt and Sorokin 2009). Entry of GBCAs from the intravascular circulation into the CSF compartment has been demonstrated in both humans and rats. Gadolinium has been detected by ICP-MS in the CSF of patients administered gadobutrol and is detectable from as early as 1.1 h and up to 24 days later (Nehra et al. 2018a, b). Moreover, CSF gadolinium has been detected after a lumbar puncture after i.v. gadobutrol administration. The clearance from the CSF demonstrated first-order kinetics and gadolinium was detectable up to 30 days post-administration (Nehra et al. 2018a, b)**.** T2 fluid‐attenuated inversion recovery (FLAIR) in patients also shows an increase in signal intensity in the CSF from 3 h and is still noticeable up to 24 h post-administration (Bozzao et al. 2003; Deike-Hofmann et al. 2019). These data suggest a potential route from the blood to the CSF; however, the mechanism by which GBCAs cross the blood–CSF barrier remain unknown and there are differences between transport through the choroid plexus and the BBB. Normal transport mechanisms include diffusion through influx (i.e. Ca^2+^ transport), efflux (e.g. iodide), vesicles (e.g. folate) or diffusion (e.g. water) (Taylor and Brown 1943; Redzic 2011; Johanson 2018). It is possible that GBCAs and also soluble gadolinium bound to macromolecules (e.g. ferritin) are sequestered in the choroid plexus (Strzeminska et al. 2021). CSF allows distribution into the subarachnoid space, but a significant portion also drains to lymph nodes. Indeed intrathecal administration of GBCAs in rats shows uptake in cervical lymph nodes, lymphatic vessels and nodes, and intravenous administration of GBCAs demonstrate enhancement of nodal metastases (Klerkx et al. 2010; Müller et al. 2017; Eide et al. 2018). Once in the subarachnoid space GBCAs may enter the brain parenchyma through the perivascular system and the pial–glial basement membrane (Morris et al. 2016; Fingerhut et al. 2018). Electron dense gadolinium foci have been found in the capillary basement membranes, further suggesting they can enter the brain in the presence of an intact BBB (McDonald et al. 2017b; Smith et al. 2017; Rasschaert et al. 2018b). This raises the possibility that the gadolinium derives from the blood and is transported across the BBB. As with the mechanism of gadolinium retention, its clearance is also poorly understood. In the absence of transmetalation of GBCAs it is possible the intact chelate is cleared through the intramural periarterial drainage (IPAD) pathway from the brain, through the drainage from basement membranes of the vasculature (Rasschaert et al. 2020). However this clearance mechanism is rapid and could only contribute to clearance of GBCAs in the acute period following administration, the long-term clearance mechanisms are not yet elucidated (Aldea et al. 2019).

## Toxicological effects of gadolinium-based contrast agents

The acute toxicity of rare earth elements including gadolinium, have been expansively researched in recent decades (Nemery 1990; Hirano and Suzuki 1996; Kuo 2008a; Pagano et al. 2015). Whilst acute toxic effects may be as a result of gadolinium interference with calcium-dependent processes, human data are limited (Tweedle et al. 1995; Hirano and Suzuki 1996). GBCAs are very well tolerated at clinically relevant doses as a result of the Gd^3+^ being chelated and not free or available. Whilst toxicity is seen at supraclinical doses of GBCAs animals, there are no clinical data to support any clinical relevance of these preclinical findings (Vogler et al. 1995). Preclinical studies evaluating safety pharmacology, toxicology, genotoxicity, local tolerance, and reproductive and developmental toxicity have supported their approval by medical agencies, worldwide. However, the standard preclinical safety requirements may not be able to detect nuanced, rare or subtle effects which raises the demand of developing novel approaches such as animal models of disease or improved assay sensitivity (McDonald et al. 2018)**.**

### Toxicological effects on skin in humans with normal renal function

Cases of gadolinium retention in the skin with clinical sequelae are rare in patients with normal renal function; therefore, data must be assessed with caution. Extremely high, cumulative doses (61 CE-MRI over 11 years) of GBCAs in a patient with normal renal function did lead to a significant concentration of gadolinium in the skin (14.5 ± 0.4 μg/g); however, no macroscopic changes were reported (Roberts et al. 2016b). There have been 3 cases of patients who have been identified as having gadolinium associated plaques, which were present in the absence of renal disease. These plaques showed dermal fibrosis and eosinophilic, collagenous, or sclerotic bodies in various stages of calcification, and may be associated with pruritus; however, gadolinium concentrations within these plaques has not been measured (Gathings et al. 2015; Olayiwola et al. 2019). Nevertheless, more studies are needed to fully understand any possible pathologies associated with gadolinium retention in the skin of patients with normal renal function. Although linear GBCA administration is contraindicated in patients with impaired renal function (as measured by glomerular filtration rates), there is a need to fully record total cumulative doses of each GBCA administered.

### Toxicological effects on skin in animals

Given the association of GBCA administration with NSF there has understandably been a focus on examining the link between skin retention of gadolinium and pathophysiological effects. We have discussed the retention of gadolinium in various organs after repeated administration of GBCAs, but it is important to understand this in context and the physiological effects of any retention. Skin lesions after GBCA administration and where severity correlates with gadolinium retention have been reported in nonclinical studies. After 80 human equivalent doses of gadodiamide administration, rats exhibited ulceration of the skin as well as fibrosis, collagen deposits, loss of extracellular space, thickening of the dermis and increased cellularity, at the histological level (Sieber et al. 2008). These skin lesions correlated with increasing gadolinium concentrations in the skin, liver, and femur. Another study showed that gadodiamide administration of over 28 human equivalent doses in rats also resulted in increased dermal cellularity (Wáng et al. 2015). Additionally, in mice with chronic kidney disease which were administered multiple doses of gadodiamide, the skin was affected by hair loss, reddening, ulceration, skin thickening and skin tightening 10 weeks post-administration (Bose et al. 2015). The extent of these lesions is associated with not only the dose, but frequency of administration. Animals were given 3 doses of gadodiamide at intervals of 24 h, 14-, 28- or 56- days. Shorter intervals between gadodiamide administration resulted in more severe skin lesions but the interval time did not affect the overall concentration of gadolinium in the skin, suggesting formation of skin lesions to be dependent on the frequency of dosing and not total dose given or skin concentration of gadolinium. Animals dosed at 24 h intervals all developed macroscopic skin changes from 3 days post-injection and were the most severe lesions compared to other groups but demonstrated some resolution on day 56 after the final injection. In the animals which had a 56-day interval between injections, 4 of 6 developed macroscopic skin lesions which completely resolved 28 days after the last injection. These skin lesions resolved in animals administered gadodiamide with long injection intervals, even though the amount of retained gadolinium was equivalent to that of animals dosed with shorter injection intervals. This lends credence to the idea that it is not the total dose, but the latency between injections which is most important in skin lesion development and that skin lesions are an acute inflammatory response to GBCAs and not due to long-term retention (Pietsch et al. 2011). This hypothesis is also supported by the acute response after administration (but before skin lesion development) of increased inflammatory markers such as transforming growth factor-beta 1 (TGFβ1) and cytokines, which was reduced 14 days post-injection. Others have also shown similar increases in inflammatory markers such as C-reactive protein and histamine (Grant et al. 2009). Whilst both macrocyclic and linear agents can lead to skin gadolinium retention, the presence of lesions after supraclinical doses has not been found with macrocyclic agents. Cumulatively, gadolinium levels in the skin after all 3 injections were similar between all interval groups, again suggesting that lesions resultant from gadolinium are related to acute exposure and not gadolinium retention, although speciation was not performed in this study so it cannot be attributed to free gadolinium (Pietsch et al. 2011). To try and recapitulate the conditions seen in patients at risk of developing NSF and to create an animal model of NSF, 5/6 nephrectomised rats were given around 80 human equivalent doses of Omniscan, magnevist, gadodiamide or GdCl_3_ (Grant et al. 2009). In nephrectomised and naïve animals both Omniscan and gadodiamide administration resulted in the development of skin lesions, with gadodiamide induced skin lesions appearing more rapidly, possibly as a result of no excess chelate to soak up free gadolinium. There was, however, no fibrosis in these lesions as evidenced by no increase in collagen density or fibroblasts, and skin thickness did not increase. These animals exhibited excessive skin scratching concomitant with the time the first lesions developed, indicating pruritus. This suggests that skin lesions developed in response to trauma rather than as a result of dermal changes. Magnevist-treated animals did not develop any skin lesions, which does not reflect the significant numbers of NSF cases seen with magnevist administration. This coupled with the lack of fibrosis and no differences between the nephrectomised and control groups, means this cannot be considered a model of NSF. A 28-day repeat dose study in cynomolgus monkeys with a maximum cumulative dose of 35 mmol/kg or 112 human equivalent doses showed no skin lesions in any animals, demonstrating species variability in development of skin lesions (Harpur et al. 1993). Gadodiamide has been shown to increase fibronectin expression in vitro in a dose and time dependant manner. However the macrocyclic gadoteridol has also been shown to increase fibronectin and whilst changes in skin thickness and cellularity are less pronounced than with gadodiamide, they are still apparent (Do et al. 2014). It is important to note that these animal studies where skin lesions are observed after multiple GBCA doses, are at doses much higher than what would be used in the clinic.

Intraepidermal nerve fibre density (IENFD) assessment allows the quantification of C-fibres in the epidermis and has in recent years been used to assess neuropathy. Mice injected with GBCAs showed a reduction in IENFD 4 weeks post-dosing with both linear and macrocyclic administration, compared to control groups, whereas only linear agents exhibited a significant increase in terminal axonal swellings (TAS) per IENFD compared to a control (Radbruch et al. 2020). These data suggest that it is the intact chelate which may be responsible for reduced IENFD; however, the clinical relevance of this is unknown as decreased IENFD and increased TAS in relation to neuropathic pain is poorly understood and studies are limited. This study is limited in several ways. Whilst IENFD was reduced with linear and macrocyclic agents, the skin concentration of gadolinium after gadodiamide administration is far higher, a difference which is not reflected in overlapping data between the treatment groups. Although the authors assessed nerve density as a biomarker of neuropathy, there was no evidence of neuropathy in these animals themselves, and no previous studies have reported pain after multiple gadoterate administrations (Robert et al. 2015, 2018; Kartamihardja et al. 2016b; Rasschaert et al. 2018b; Fretellier et al. 2019; Jost et al. 2019). The absence of supportive data suggests that these data may be an anomaly.

### Nephrogenic systemic fibrosis

NSF is a rare disease with the first recorded case in 1997 and found exclusively in patients with end-stage kidney disease. It was not until 2006 where the possible association of GBCA administration and NSF was first identified as a result of slower GBCA elimination due to impaired clearance mechanisms (Grobner 2006; Marckmann et al. 2006). NSF is predominately a disease of the skin and is characterised by skin fibrosis, and fibrosis of the subcutaneous tissues and skeletal muscle of the arms and legs. In some cases, it is possible for fibrosis to become systemic and affect other organs including the pericardium or dura mater (Sanyal et al. 2011). NSF progresses to a systemic, chronic condition and is potentially life threatening (Bhave et al. 2008; Kay et al. 2008; Sanyal et al. 2011; Bernstein et al. 2012). After skin, muscle is the most commonly involved organ with NSF and diaphragm, oesophageal and deep muscles have shown fibrosis with severe atrophy and infiltration of endomysium and perimysium with fibrous tissue, and skeletal tissue showing vascular calcification and CD34+ cellular fibrosis (Sanyal et al. 2011). Fibrosis of the muscles with involvement of the subcutaneous fascia, and striated muscles associated with thickening of tendons and peri-articular tissues has also been observed (Mendoza et al. 2006). This fibrosis extends through the lobular septa and into underlying fascia and muscle (Thakral and Abraham 2009). Whilst most gadolinium containing foci are localised to the vasculature walls, they have also been seen to be perivascular and associated with increased vascular calcification (Schroeder et al. 2008; Singh et al. 2008; Sanyal et al. 2011). Initially termed nephrogenic fibrosing dermopathy (NFD), it was renamed NSF due to the extra-cutaneous fibrosis which was detected (Cowper et al. 2001; Bernstein et al. 2012). Although there has been a spotlight on NSF given its association with GBCA use, it is a rare condition in comparison to the extensive use of GBCAs which has spanned decades. To date there are around 375 cases listed in the NSF registry, and confirmed cases are only found in patients with kidney disease who have been administered GBCAs (Abu-Alfa 2011). This registry are cases that were confirmed using rigorous methodology but does not necessarily include all NSF cases. The identification of this link has led to restriction of use in patients with severe chronic renal failure or acute renal failure. Additionally, many medical agencies have banned the use of linear GBCAs in patients with renal disease with a GFR or estimated GFR less than 30 ml/min/1.73 m^2^ (Bhave et al. 2008; Tsushima et al. 2010)**.** The early symptoms of NSF include pruritic, red patches on the skin with pain and oedema followed by joint stiffness, muscle weakness and deep bone pain. Long-term symptoms are also characterised by epidermal atrophy, follicular dimpling and hair loss (Weigle and Broome 2008; Bernstein et al. 2012). Diagnosis involves skin biopsy with microscopic analysis that shows thickened dermal collagen, increased CD34+ spindle cells and dermal mucin (positive for colloidal iron stain) (Larson et al. 2015). Other potential risk factors may include recent surgery, acidosis, hyperphosphatemia, liver disease and high and multiple doses of GBCAs (Grobner 2006; Kay et al. 2008; Mazhar et al. 2009; Elmholdt et al. 2010; Zou et al. 2011; Thomsen et al. 2013). Linear GBCAs are more liable to transmetalate and release gadolinium, and it is this free gadolinium which has been proposed to contribute to NSF evidenced by linear agents being more associated with NSF development (Kuo 2008a)**.** Conversely, the risk of NSF in stage 4 or 5 chronic kidney disease patients with macrocyclic GBCAs is estimated to be less than 0.07% (Woolen et al. 2020).

The data pertaining to the potential mechanisms of toxicity are limited and the pathophysiology of NSF is poorly understood, in part due to the low number of cases. It has been suggested that free gadolinium is released from the intact GBCA through transmetalation, and as they are excreted through the kidney, patients with low GFR have increased residence times of the GBCA, increasing the chance for transmetalation to occur (Joffe et al. 1998; Bhave et al. 2008). NSF generally occurs a short period (weeks to months) after GBCA administration, but delayed onset (years) has been noted, most notably in a patient who developed NSF 10 years post exposure to GBCA (Grobner 2006; Larson et al. 2015; Thomson et al. 2015). Fibroblasts are found in early NSF lesions and the accumulation of activated fibroblasts increases with lesion duration, as does macrophage number, concomitant with increased TGFβ1 (Cowper et al. 2000; Swartz et al. 2003; Jiménez et al. 2004; Newton and Jimenez 2009). Fibrocyte markers are low in expression in early NSF but increase with advancing disease (Cowper et al. 2000, 2001). It is thought that the ability of fibrocytes to endocytose surrounding material, may contribute to NSF mechanisms and progression (Kuo 2008b). It is currently thought that it is dechelated or free gadolinium which results in toxicity in the case of NSF. This is based on increased elimination half-lives in patients with renal insufficiency and thus increased residence time, differences in physicochemical properties, presence of gadolinium in macrophages, bone and in the lesions of NSF patients and association of a single high dose, rather than a cumulative dose, with NSF (Idée et al. 2006; White et al. 2006; Collidge et al. 2007; High et al. 2007; Prince et al. 2008). There are, however, phenomena which are not explained by this proposed mechanism such as the very small cases which have been observed in patients with renal insufficiency, gadolinium presence not always found in NSF biopsies and similarity in conditional stability constants at physiological pH (High et al. 2007; Kuo et al. 2007; Prince et al. 2008). In vitro assays have also demonstrated inflammatory and cytokine release. GdCl_3_ can increase the expression of cytokines including, IL-6, IL-13, IL-4, TGF β1 in cultured human peripheral blood monocytes (PBMC), but gadodiamide and gadopentetate were also shown to activate cytokines (Wermuth et al. 2009). GBCA addition to human fibroblast, monocyte and macrophage cultures can result in acute profibrotic and proinflammatory responses, suggestive of an effect of the organic complex, rather than gadolinium itself (Bhagavathula et al. 2009; Del Galdo et al. 2010). Given the association of macrophages in fibrotic disease and their presence in NSF, this suggests a mechanistic link between intact GBCAs and NSF (Jiménez et al. 2004; Thakral and Abraham 2009; High et al. 2007). Fibroblasts cultured from NSF lesions from patients exposed to Omniscan demonstrate increased hyaluronan and collagen and Omniscan, Magnevist, MultiHance and ProHance all increased fibroblast proliferation in a human dermal fibroblast cell line (Edward et al. 2008; Varani et al. 2009). These data may suggest an alternative mechanism of GBCA effects due to increased residence time of GBCAs in renal insufficiency, high GBCA concentrations potentially initiating inflammatory and fibrotic responses, and phagocytosis of GBCA. What is clear is that NSF is a rare, complex condition that requires several factors to converge within a patient to instigate the development of this pathology. Renal failure and an increase in total exposure to GBCAs is certainly a key factor, together with others that may include certain coincident pathologies, a pro-inflammatory background and/or poorly defined genetic factors.

Currently, there are no viable, effective treatments for patients with NSF. Dialysis has been used in an attempt to remove gadolinium from the blood, but there is no evidence to suggest that this may have an impact on NSF development (Rodby 2008).

### Gadolinium deposition disease

Whilst gadolinium retention in the bone and other tissues of patients even without renal disease has been widely reported and studied, the majority of evidence suggests that this is not causally associated with any clinical symptoms (Kanal 2016; Kanda et al. 2017; Olchowy et al. 2017; Tedeschi et al. 2017; Pullicino et al. 2018). One group has suggested the existence of a condition they have coined “gadolinium deposition disease” (Semelka et al. 2016b). This group recruited 50 subjects with no reported evidence of renal dysfunction, using an online survey to assess their self-reported symptoms (Burke et al. 2016). Participants had received an average of 4.2 doses of GBCAs including gadobutrol, gadopentetate, gadobenate, gadodiamide, gadoversetamide, multiple agents or unknown agents. Subjects reported symptoms including bone/joint pain, head/neck pain, flu-like symptoms, skin changes, digestive symptoms, chest symptoms, generalised whole-body symptoms, or other pain. In a similar study, the same group surveyed a further 42 people from online gadolinium toxicity groups, with no reported evidence of renal dysfunction (Semelka et al. 2016b). All participants had detectable gadolinium in the urine extending 1 month post GBCA (gadodiamide, gadobenate, gadoversetamide, gadobutrol or unknown) administration, and some had increases in the thyroid (*n* = 1), scalp (*n* = 1), skin (*n* = 2) and hair (*n* = 2). These participants described pain that manifested a central, peripheral, headache, bone, or other sites, in some cases lasting more than 3 months and described this as sharp pain or intense burning. Skin thickening was seen in 22 participants and skin discolouration in 28, with 29 subjects reporting “clouded mentation” which lasted more than 3 months. Whilst the majority of subjects in these studies were given linear agents, at least 2 were administered a macrocyclic agent, which are indicated in renal insufficiency patients and exhibit much reduced gadolinium retention. This study is extremely subjective as it relies on self-reporting and no tangible measurements of any disease. In the brain, gadolinium retention has been shown to be predominantly in the dentate nucleus and globus pallidus. However, these patients did not experience disorders in movement but far more generalised symptoms, which would not be anticipated to occur with any pathology in these brain regions. As others have remarked, this makes it difficult to prove a link between GBCA administration and these collection of symptoms, especially given the selection bias and absence of clinical history to explore other diagnoses (Layne et al. 2020). The existence of gadolinium deposition disease is also questioned by many in the field citing the lack of evidence to suggest that gadolinium deposition disease exists. These conclusions arise from the lack of well-designed clinical trials, development of clinical sequelae with normal renal function and the lack of link between symptoms and gadolinium levels (Lyapustina et al. 2019; Layne et al. 2020). More recently a study in over 1000 patients administered gadodiamide or gadoterate were assessed to examine the new-onset symptoms compared with an unenhanced MRI control group (Parillo et al. 2019). Some patients reported some symptoms identified by Semelka et al. to constitute GDD, namely fatigue and mental confusion after GBCA administration. With gadodiamide CE-MRI 12.4% of patients reported GDD-like symptoms and 12.3% with gadoterate; however, 6.6% of patients reported the same symptoms in the absence of any GBCA. There was no difference in other alleged GDD symptoms between the control group and GBCA administered groups and symptoms reported were mild with resolution by 24 h after MRI. This study, however, did not account for comorbidities between groups given CE-MRI or unenhanced MRI. In a recent study by Layne et al., patients who requested assessment for potential gadolinium toxicity were assessed by a clinical toxicologist and patient samples were analysed. Patients received at least 2 GBCA administrations and had significant comorbidities. Gadolinium was detected in the whole blood, plasma, and urine in 69%, 77% and 95%, respectively, and concentrations in individual patients were positively correlated between different samples. The authors concluded that there were no clinical features of toxicity associated with GBCA use (Layne et al. 2021).There exists a huge array of preclinical studies that use high and repeated doses of GBCA administration, and none have shown strong evidence of toxicity associated with normal renal function. Whilst it is much harder to assess generalised pain in response to GBCA administration in animals, there have been no reported signs indicative of generalised pain in animals.

Since their approval, over 500 million doses of GBCAs have been administered worldwide and self-reported cases of GDD are comparatively negligible (McDonald et al. 2018). Given the high usage it might be expected that many more patients would have occurrences of GDD if there was a causative effect correlated to GBCA usage and importantly, the existence of GDD is still not proven.

### Nephrotoxicity

Association of GBCA administration with nephrotoxicity in humans is very limited. One study detailed an acute nephrotoxic effect after GBCA administration where a 56-year old woman with normal renal function had 2 GBCA administrations and within a few days developed acute renal failure with a biopsy showing acute tubular necrosis (Akgun et al. 2006)**.** In patients exposed to i.v. GBCAs with a serum creatinine of 208 (106–318) µmol/L, GBCA-associated nephrotoxicity occurred in 0–5% of cases (Perazella 2009). Upon intra-arterial administration in patients with a serum creatinine of 265 (229–353) µmol/L, GBCA-associated nephrotoxicity occurred in 5.3–50% of the population, although many of these patients had stage 3–5 chronic kidney disease.

Although gadolinium can be retained in the kidneys after GBCA administration, there is a concern that reduced clearance of GBCAs by the kidneys due to renal dysfunction, could result in increased residence time of GBCAs in the body and subsequent higher retention of gadolinium. Proximal tubular vacuolation has been demonstrated in animals treated with GBCAs (Elmståhl et al. 2007; McDonald et al. 2017b; Do et al. 2020). This vacuolation phenomenon presents as lipid rich vacuoles with electron dense structures, with gadolinium deposits lining the lipid deposits in the renal proximal tubule (Do et al. 2020). Proximal tubular vacuolation is a common finding after administration with high volume contrast agents (iodinated and GBCA) and hypertonic solutions (Simon et al. 1964; Battenfeld et al. 1991; Harpur et al. 1993; Döhr et al. 2007). This occurrence was shown to be as a result of transient storage of the contrast agent and not associated with functionally significant impairment of tubular or cellular processes, with resolution over time (Dobrota et al. 1995; Morcos et al. 1996; Wack et al. 2012). Renal function impacts the clearance of GBCAs, and this is relevant to the issue of NSF in patients with impaired renal function. Although creatinine clearance is the most widely used measure of kidney function across human and animal studies, it has significant limitations in terms of sensitivity and specificity. A few studies have shown that supraclinical doses of linear GBCAs can impact creatine clearance in rats, concomitant with tubular vacuolation, which can be attenuated with hydration (Brillet et al. 1994; Chien et al. 2012).

### Toxicological effects on the central nervous system

As discussed, there is currently no evidence for brain toxicity as a result of standard GBCA administration, although it is possible for there to exist more subtle behavioural abnormalities in the absence of overt toxicity. There have been no widespread reports of CNS toxicities reported in humans.

To examine the behavioural effects of GBCA on development, pregnant mice were administered high doses of gadoterate or gadodiamide during gestational days 15–19 and offspring were tested for behavioural effects from 70 days postpartum (Khairinisa et al. 2018). Assessment of locomotion and anxiety in an open field test showed no effect on travelling distance in males compared with controls but gadodiamide resulted in less time spent in the centre. Conversely, females spent less time travelling but only spent less time in the centre zone in one, 10-min time bin. Although increased grooming behaviour is known to impact performance in open field tests, the authors did not comment on skin lesions or the grooming which may be resultant (Habermeyer et al. 2020). Motor coordination as measured by the rotarod test showed both male groups of GBCAs differed from the control but were very similar to each other even though the gadodiamide group had 71 times more gadolinium in the brain compared with gadoterate. Similarly, the grip strength test found both GBCAs resulted in worse performance in males but was only seen in females in the gadodiamide group. Although males showed a significant difference with gadoterate that was not seen in females, the females had 5.7-fold more gadolinium in the brains than the males. To assess spatial memory and discrimination using object recognition and object in location, both GBCA groups showed similarly worse performance for both sexes, even though the levels of brain gadolinium ranged from 8.2 (gadoterate) to 618 ng/g (gadodiamide). In this test, females performed worse with both agents, but only the males were affected in the gadodiamide group. A nociceptive test (Von Frey) again showed sex differences with no effect in females but a lower nociceptive threshold for both GBCA groups. This highlights the potential sex differences but also that gadolinium concentration does not correlate to behavioural changes after supraclinical GBCA administration in mice. Other studies have shown no effect with linear agents on open field or rotarod tests, using similar dosing regiments in rats (Bussi et al. 2018b; Habermeyer et al. 2020).

In neonatal and juvenile rats given up to 15 mmol/kg gadobenate there was no effect on behaviour or cognitive function (Bussi et al. 2018b). To assess behaviour, the authors employed the Morris water maze to assess memory, the open field test for locomotion and anxiety and a functional observation battery for gross functional assessment, and no significant differences were seen for any group. Rats have been shown to perform worse in heat and mechanical hyperalgesia tests, where animals treated with supraclinical doses of gadodiamide, but not gadoterate, had reduced withdrawal latency, suggesting they may feel more pain (Alkhunizi et al. 2020). In line with this, more gadolinium was found in the peripheral nerves with gadodiamide than gadoterate. Fretellier et al. (2019) also examined behavioural effects in juvenile or adult rats treated with 20 human equivalent doses of gadodiamide or gadoterate. There were no differences in the elevated plus maze, which corroborates existing literature which shows no hippocampal morphological changes or impairments in hippocampal neurogenesis, an area of the brain important in anxiolytic response and memory (Smith et al. 2017; Alkhunizi et al. 2020; Davies et al. 2021). A T-maze is also a measure of hippocampal function and repeated dosing of gadodiamide and gadoterate revealed no significant changes compared to a control group (Alkhunizi et al. 2020). In the balance beam test, gadodiamide resulted in significant reduction in scores compared to gadoterate and control groups; however, this was as a result of male animals not completing the test and not as a result of a worse performance, and the authors themselves conclude that there were no significant treatment related effects. Additionally, the authors found no histological abnormalities in the cerebellum and treatment did not affect the size of litters. Habermeyer et al., found no effect on gait analysis or pre-pulse inhibition but did find a reduction in transient startle response with gadodiamide low and high doses (4.8 and 14.4 mmol/kg), which was not seen with intermediate doses or at all time points, suggesting reversible and transient effects. No gadolinium was detected in the cochlear root nucleus, but the primary startle pathway consists of the auditory nerve; however, no hearing deficits were observed. The authors noted that gadodiamide-treated animals exhibited more grooming behaviour in the open field test, associated with skin lesions and they found no effect on neuronal numbers or pathway deregulation, suggesting no observable brain toxicity (Habermeyer et al. 2020). Whilst these behavioural findings are important to consider, it must be noted that these are performed using supraclinical doses of GBCAs and there has been no preclinical evidence of chronic toxicity or histopathological changes in the brain in vivo as a result of gadolinium retained in the brain after GBCA administration (Lohrke et al. 2017; McDonald et al. 2017b; Smith et al. 2017). Recently, in a very comprehensive assessment of motor and behavioural function, mice were administered approximately 33 human equivalent doses of gadoteridol or gadodiamide (Akai et al. 2021). To assess these parameters the tests used were rotarod, open field test, elevated plus maze, light–dark anxiety, locomotor activity, passive avoidance, Y-maze and forced swim test. This combination of tests evaluates motor coordination, locomotion, anxiety, exploration, circadian rhythm, depression and long and short-term memory. There were no differences between any of the groups in the numerous parameters analysed. Although not directly comparable due to the use of adult mice and not offspring, this comprehensive study is in contrast to Khairinisa et al. (2017), which showed some effects in an open field test, rotarod and object recognition (Khairinisa et al. 2018). It also supports the many other studies which have looked at the potential effect of GBCA administration behaviour (Bussi et al. 2018b; Fretellier et al. 2019; Alkhunizi et al. 2020; Habermeyer et al. 2020).

### Acute hypersensitivity reactions

Another possible effect of GBCA administration is the presentation of an acute hypersensitivity reaction. Allergic reactions in response to GBCA have been well documented though the risk is relatively low in comparison to other contrast media for example iodinated agents (Murphy et al. 1996, 1999; Dillman et al. 2007; Abujudeh et al. 2010; Prince et al. 2011; Morgan et al. 2011; Davenport et al. 2013; Okigawa et al. 2014; Bruder et al. 2015; Fakhran et al. 2015; Power et al. 2016; Granata et al. 2016; Sodagari et al. 2018)**.** Jung et al. examined the occurrence of acute hypersensitivity reactions in patients who had received GBCA injections over a 6-year period. They identified 102 patients who exhibited hypersensitivity reactions, and patients who had a history of episodes of hypersensitivity reactions with GBCAs had an increased rate of reoccurrence with subsequent administration (Jung et al. 2012). Among these patients, the GBCA with the smallest risk of immediate hypersensitivity reactions was gadodiamide (0.013%) and gadobenate had the highest risk (0.22%). Other risk factors included being female, allergies and asthma. The overall risk of hypersensitivity reactions with GBCA was determined to be low (0.079%), but the reoccurrence was 30% in patients with a history of GBCA acute hypersensitivity reactions (Jung et al. 2012). A retrospective analysis demonstrated immediate allergic-like reactions to be 0.2, 0.5, 1.2 and 3.3 per 1000 administrations of the GBCAs gadodiamide, gadopentetate, gadobenate, and gadoteridol, respectively (Prince et al. 2011). In this analysis gadobenate resulted in more severe reactions, with 3 patients becoming unresponsive, and one death. Between 2004 and 2009 in the USA, the incidence of deaths unconnected to a diagnosis of NSF was 0.15, 0.19, 0.97, 2.7, and 0.7 per million doses for gadodiamide, gadoversetimide, gadopentetate, gadobenate, and gadoteridol, respectively. Additionally, a retrospective study of over 150,000 patients confirmed these findings, with gadobenate and gadobutrol having the highest rates of allergic-like reactions and gadodiamide the lowest incidence of reactions. Allergic reactions requiring hospitalisation occurred in 3 gadobutrol and 3 gadobenate administrations (McDonald et al. 2019). A meta-analysis of over 700,000 GBCA administrations across 9 studies demonstrated similar findings, with the difference in likelihood of reactions being linked to the type of GBCA (Behzadi et al. 2018). The non-ionic linear GBCA gadodiamide had the lowest rate of immediate adverse reactions (1.5 per 10,000 administrations) which was significantly less than that for linear ionic GBCAs (8.3 per 10,000 administrations) and less than that for non-ionic macrocyclic GBCAs (16 per 10,000 administrations). The overall severity of these reactions was 81%, 13% and 6% categorised as mild, moderate, or severe reactions respectively, and when only moderate and sever reactions were considered, non-ionic linear GBCA had a lower relative risk compared with non-ionic macrocyclic GBCAs. These data describe another dynamic to the pharmacological profile of different GBCAs. Gadolinium retention, which is commonly seen but without clinical effect, is lower with macrocyclic GBCAs, but conversely acute reactions, which are rare but can be severe, are lower with non-ionic linear GBCAs. Considering this, like all drugs GBCAs have a certain risk benefit profile that should be balanced depending on the needs of the patient, for example it may be more appropriate to use a GBCA with a lower level of gadolinium retention if repeat administration in a younger patient is indicated, or a GBCA with a lower incidence of allergic-like reactions in an older patient, perhaps with a history of allergic reaction, that may be more vulnerable to such effects.

### Other observations

Other risk factors are not well understood, including potential susceptible patient populations for example foetal, paediatric or pregnant patients (McDonald et al. 2018). Paediatric patients who had received hematopoietic stem cell transplants who had undergone GBCA CE-MRI with the macrocyclic agent gadoterate, all had a positive correlation between total GBCA dose and gadolinium and iron concentration in the liver. A lack of clinical data demonstrates the need for more research in different patient populations. In 397 pregnant woman who underwent CE-MRI with GBCAs there was an association of inflammatory, rheumatological, infiltrative skin conditions and an increased risk of stillbirth and neonatal death, compared with pregnant women who did not undergo MRI or CE-MRI. However, this study has a very small subset of patients and by the authors own admission, the study was underpowered and multiple comparisons may have led to statistical errors. Additionally the indications for the MRI were not recorded meaning it is likely that underlying health conditions differed between the GBCA exposed and non-exposed populations and could have influenced pregnancy outcomes, unrelated to GBCA administration (Ray et al. 2016).

## Chelation therapy in humans and animals

Chelation therapy has been used for decades to treat heavy metal intoxication and involves a chemical chelating agent to bind free, metal ions to facilitate elimination from the body (Sears 2013). Thus, the possibility of chelating agents to eliminate retained gadolinium has been explored. Rats given 2 human equivalent doses of gadodiamide or gadobutrol were also treated with Ca-DTPA and ICP-MS analysis was performed to measure gadolinium concentration. Ca-DTPA increased urinary excretion of gadolinium from 10 to 114 nmol in animals given gadodiamide and also reduced brain gadolinium retention. With gadobutrol administration there was no effect on urinary excretion or brain concentrations as the basal clearance was already higher (Boyken et al. 2019). The overall concentration of gadolinium in the bone and skin did not vary with chelation therapy, possibly due to the relatively high concentrations (~ 2700 nmol and 500 nmol in the bone and skin, respectively). The chelator, Ca-DTPA follows a similar distribution to GBCAs and is found in the extracellular space and is unable to enter cells, thus chelation is limited to blood and extracellular spaces. Deferoxamine is available as a commercial chelation therapy, but in rats has shown no impact on gadolinium retention (Oh et al. 2020). In humans however, one study has shown that chelation with deferoxamine had a significant reduction (52.1–99.8%) in the gadolinium liver concentration (Maximova et al. 2016). Biodistribution studies in mice using an isotope of gadolinium demonstrated the ability of 3,4,3-LI(1,2-HOPO) when administered prophylactically or after administration of ^153^Gd to chelate and reduce gadolinium retention (Rees et al. 2018)**.** Caution must be exercised with chelation therapy due to the ability of these chelates to bind endogenous metals like zinc and manganese. Human data is very limited with regard to chelation therapy. Blaurock-Busch et al. performed a study in patients to evaluate the effectiveness of chelation therapy and found that it had no effect on gadolinium elimination (Blaurock-Busch 2019)**.** Additionally, patients who were deemed to have “gadolinium deposition disease” were administered Ca-DTPA or Zn-DTPA to chelate retained gadolinium and urinary gadolinium was measured (Semelka et al. 2018). These patients had a 30-fold increase in urinary gadolinium when treated with chelation therapy in monthly intervals, and in patients with weekly intervals, there was a 12.9-fold increase. Thirteen of 25 patients reported an improvement in symptoms, 10 were unchanged and there was worsening in 2 patients. This study, however, did not determine which reservoirs the gadolinium was being removed from and was in a very small sample set. Despite this finding no extensive, controlled studies have been performed to fully assess the utility of chelation therapy after GBCA administration, which may even be harmful due to chelation of endogenous metals. It is important to note that there is no consensus on the existence of gadolinium deposition disease, and in patients with normal renal function where there are no gadolinium related toxicities, chelation therapy would likely have negligible effects.

## Summary and conclusions

Following decades of use, the short-term pharmacokinetics of GBCAs have been well described in many studies. All GBCAs show rapid distribution throughout the blood and extravascular space with subsequence rapid clearance by the renal route. The exceptions are gadoxetate and gadobenate which have a proportion of hepatobiliary excretion (50 and 0.6–4%, respectively, in humans), enabling liver imaging (Weinmann et al. 1991; Hamm et al. 1995; Lorusso et al. 1999; Spinazzi et al. 2003; Bayer HealthCare Pharmaceuticals 2014). Since GBCAs are excreted predominantly through the renal system, the in vivo residency of the contrast agents is significantly increased in patients with impaired renal function. All GBCAs have been shown to release a limited amount of gadolinium under certain conditions in vitro (e.g. through transmetalation of the Gd ion with other physiological ions such as zinc), and this process probably occurs in vivo to some extent, which may be one contributing factor to the detection of gadolinium in tissues such as the skin, bone and brain a long time after GBCA administration in humans and animals, alike (Darrah et al. 2009; McDonald et al. 2018; Le Fur and Caravan 2019; Davies et al. 2021; Rudnick et al. 2021). Although observed in animals and humans for all GBCAs, gadolinium retention is generally more prominent for the linear class of agents than the macrocyclic class. Human data is complicated by both a frequent uncertainty of the GBCA history of patients (both on the amount of contrast given as well as the specific agent used on each occasion) and the limited methods of analysis available. Animal studies have provided useful understanding of the long-term excretion of GBCAs and the retention of gadolinium, although it is limited considering the differences in physiology to humans and the acute nature of the studies (e.g., repeat daily administration of high doses of GBCA, which can aid quantification), which does not reflect clinical practice. However, even animal studies have yet to be able to fully determine the nature of the gadolinium retained (whether intact within the parent GBCA, insoluble metal ion or whether conjugated to endogenous proteins or molecules). More recent animal studies are starting to uncover possible mechanisms to explain how gadolinium may enter the brain.

GBCAs, used in large volumes and for decades with no indications of any associated toxicity, required further scientific investigation following the identification of NSF (a very rare but potentially fatal outcome of GBCA use in a small percentage of patients with renal failure) and of gadolinium retention in the brain (a more common occurrence following repeated GBCA administration, but with no identified toxicological sequelae). The development of NSF is exclusively limited to certain patients with renal insufficiency associated with increased residence time and total GBCA exposure, together with other poorly defined, idiosyncratic contributing factors. Repeated administration of linear agents results in skin lesions in rats, which are not related to the level of retained gadolinium and appear to be an acute response to closely timed, high doses of GBCA linked with pruritus and excessive scratching. Whilst this observation in rats has little relevance to the clinical situation, a different effect on the skin termed gadolinium associated plaques has been reported in an very small number of cases (3 patients) (Sieber et al. 2008; Gathings et al. 2015; Olayiwola et al. 2019). The majority of animal studies have found no effect of GBCA administration on behavioural assessments and this is in agreement with the lack of neurological sequelae in patients (Bussi et al. 2018b; Fretellier et al. 2019; Habermeyer et al. 2020; Izabela Strzeminska et al. 2021; Akai et al. 2021). Whilst few animal studies have shown behavioural effects associated with supraclinical doses of GBCAs, there is no association with gadolinium concentration, and effects have been noted in both linear and macrocyclic GBCAs (Khairinisa et al. 2018).

GBCAs remain an extremely valuable tool for the diagnosis of a large number of diseases with approximately 500 million doses administered since the 1980s (McDonald et al. 2018). As a class of related compounds, GBCAs remain well tolerated with few side effects. NSF has been effectively controlled by restrictions on GBCA use in patients with renal failure and, although investigated thoroughly, no toxicological consequence of gadolinium brain retention has yet been found.

Critical in assessing any potential toxicities as well as new opportunities in GBCA use (such as imaging of the glymphatic system) has been the combination of clinical observation and extensive human used with the wealth of non-clinical studies. This partnership will only continue to increase the understanding of this important class of compounds that remain a key pillar of clinical diagnosis of a wide variety of clinically important pathologies that would otherwise go undetected.
